# Fare to the Meeting of the American Medical Association

**Published:** 1869-04

**Authors:** M. B. Atkinson


### Fare to the Meeting of the American Medical Association.

The following arrangements have been made for attendance on the American Medical Association, May 4, at New Orleans:

The great Southern Mail Route will carry delegates to New Orleans and return by excursion tickets from New York, for $70. Tickets must be bought in New York at 229 Broadway. Time, 84 hours.

Excursion Tickets from Philadelphia by the same route *via* the Philadelphia, Wilmington and Baltimore R. R. to New Orleans, will be sold *only* by the *Permanent Secretary* for $66. Time, 80 hours. Those desiring to go by this route must notify the Permanent Secretary immediately. Permission is given to stop at any point and resume at pleasure.

The East Tennessee and Georgia and the East Tennessee and Virginia R. R. will issue excursion tickets at half fare over their roads only.

The Mobile and Ohio, the Louisville and Nashville, and Memphis and Louisville, the Selma, Rome and Dalton, the Lexington and Louisville, the South Carolina and the North Eastern, the Richmond, Fredericksburg and Potomac, Richmond and Petersburg, the Petersburg, the Virginia and Tennessee, the Mobile and Montgomery, the Montgomery and West Point, and the Milwaukee and St. Paul R. R. will return free on certificate of the Permanent Secretary.

STEAMERS.—The Steamship Line from Philadelphia will carry *via* Havana, meals included, for $100 the round trip, or $50 either way.

The Memphis and St. Louis Packet Co. will carry to Memphis or Vicksburg by boat, and balance by rail, for $20 either way, meals on boat included, or $17 and pay for meals.

The Mobile and New Orleans Steamers will issue excursion tickets at half fare.

Dr. Hibberd's circular tells all else, *so far*.

Any additional communications will be furnished you as soon as received.

Truly yours, M. B. ATKINSON.

MARCH 22, 1869.

---

The following information is communicated by the Sub-Committee of Arrangements of the American Medical Association for this city and Brooklyn:—Rate of passage per New York and New Orleans Steamer, of Livingston, Fox & Co. (office, 88 Liberty st.) *via* Havana, to a delegate of American Medical Association, either way, will be $40; rate of passage to New Orleans *via* Savannah (between S. & N. O. by rail) $48.50. By former route a steamer will leave April 24, by latter April 27.

A company of six or more delegates can each obtain from Mr. J. B. Yates, basement, 229 Broadway, Railroad Excursion Tickets from New York to New Orleans and New Orleans to New York for $70.

Time of passage by first route, 8 or 9 days; by second route, 5½ days; by last route, 3½ days.—*Medical Gazette.*

